# Symbiosis limits establishment of legumes outside their native range at a global scale

**DOI:** 10.1038/ncomms14790

**Published:** 2017-04-07

**Authors:** Anna K. Simonsen, Russell Dinnage, Luke G. Barrett, Suzanne M. Prober, Peter H. Thrall

**Affiliations:** 1 Commonwealth Scientific and Industrial Research Organization (CSIRO), Land & Water, Clunies Ross Street, Acton, Australian Capital Territory 2601, Australia; 2 Research School of Biology, The Australian National University, 116 Daley Road, Acton, Australian Capital Territory 2601, Australia; 3 CSIRO Agriculture and Food, Canberra, Clunies Ross Street, Acton, Australian Capital Territory 2601, Australia; 4 CSIRO Land & Water, Private Bag 5, Wembley, WA 6913, Australia

## Abstract

Microbial symbiosis is integral to plant growth and reproduction, but its contribution to global patterns of plant distribution is unknown. Legumes (*Fabaceae*) are a diverse and widely distributed plant family largely dependent on symbiosis with nitrogen-fixing rhizobia, which are acquired from soil after germination. This dependency is predicted to limit establishment in new geographic areas, owing to a disruption of compatible host-symbiont associations. Here we compare non-native establishment patterns of symbiotic and non-symbiotic legumes across over 3,500 species, covering multiple independent gains and losses of rhizobial symbiosis. We find that symbiotic legume species have spread to fewer non-native regions compared to non-symbiotic legumes, providing strong support for the hypothesis that lack of suitable symbionts or environmental conditions required for effective nitrogen-fixation are driving these global introduction patterns. These results highlight the importance of mutualisms in predicting non-native species establishment and the potential impacts of microbial biogeography on global plant distributions.

## Abstract (web summary)

Symbiosis with nitrogen-fixing bacteria (rhizobia) aids the growth of many legume species, but may also restrict their ability to colonize new regions lacking suitable rhizobia. Here, the authors show that symbiotic legumes are indeed less likely to become established in new regions than their non-symbiotic relatives.

Species must overcome multiple barriers to successfully establish in a novel range^1^. One such barrier is associated with mutualistic interactions, which are predicted to limit invasion success because the new range may not contain effective mutualist partners required for initial establishment^2^. Legumes (*Fabaceae*) are a globally distributed and highly diverse family of flowering plants, many of which are dependent on symbiotic nitrogen-fixing bacteria for growth and reproduction^3^. Anthropogenic activity has introduced legumes into new regions and continents at an unprecedented global scale^4^. However, it remains untested whether legume dependency on symbiotic nitrogen fixation has facilitated or hindered establishment into novel ranges.

Legume hosts acquire rhizobial symbionts horizontally via the environment. This could limit legume establishment following long distance dispersal by reducing access to compatible symbiont partners^5^,^6^ or suitable environmental conditions for efficient nitrogen fixation^7^. On the other hand, symbiotic nitrogen fixation has been purported to facilitate legume colonization, especially in disturbed or degraded habitats^8^,^9^, potentially favouring the establishment of symbiotic nitrogen-fixing legumes over non-symbiotic legumes. According to these contrasting hypotheses, symbiotic nitrogen fixation could either impede or promote plant establishment in new ranges. However, we currently lack a global macro-ecological analysis to support or refute the generality of either of these claims.

Morphological and molecular evidence show that legumes (*Fabaceae*) form one large monophyletic group and that rhizobial symbiosis evolved from a single origin over 59 million years ago^10^,^11^. This origin was followed by multiple gains and losses of the ability to symbiotically fix nitrogen across multiple clades^10^,^12^, allowing us to directly compare the relative prevalence of symbiotic and non-symbiotic legume species in non-native areas. Using an expert-annotated global legume distribution database^13^ and the most comprehensive list of nitrogen-fixing trait data available^10^, we evaluated differences in recent establishment in introduced areas between symbiotic and non-symbiotic legumes (see Methods).

In total, our data set comprises 3,213 symbiotic species and 317 non-symbiotic species. Each species record consists of its symbiotic nitrogen-fixing status and a broad characterization of its global range distribution (as shown by a list of geographic polygons, referred to as ‘regions' hereafter, describing countries, islands or states in which the species is found), and the ‘native' or ‘non-native' designation for each geographic region (see Methods section). We found that non-symbiotic legumes have spread to a greater number of geographic areas compared to symbiotic species at a global scale, providing evidence that symbiosis with nitrogen-fixing bacteria has limited establishment of legumes into novel islands, regions and continents.

## Results

### The role of symbiosis in introduction success

**Figure 1:**
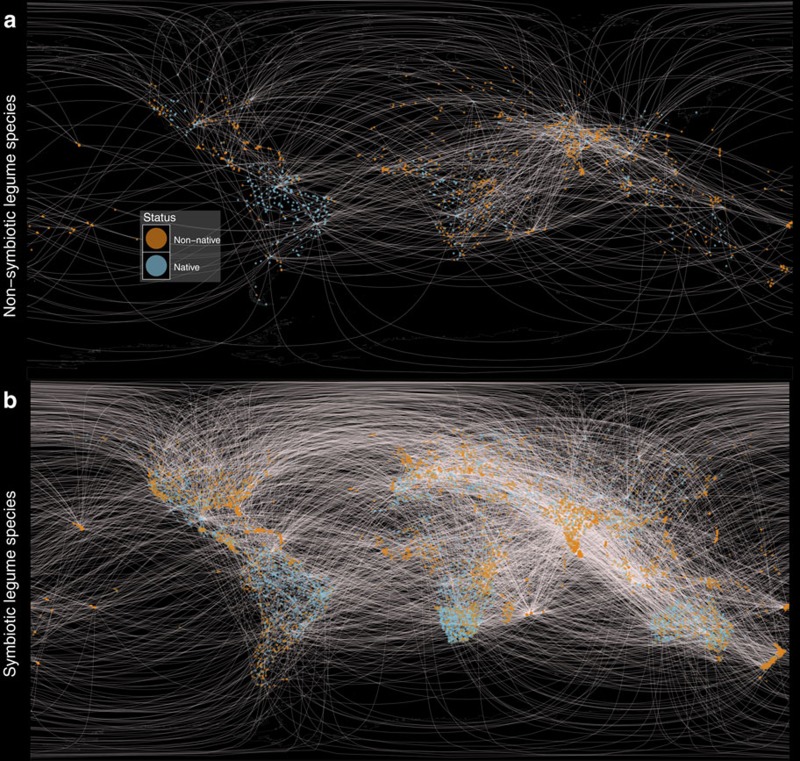
A visual representation of the network of successful legume introductions. (**a**) Non-symbiotic and (**b**) symbiotic legume species. To facilitate visual interpretation of global network patterns, each species was assigned a single native point (coloured in blue), drawn randomly from its native range, and one or more non-native points (coloured in orange) drawn randomly from each of its non-contiguous non-native ranges. Non-contiguous ranges were defined by merging all polygons whose distance was less than 5 degrees Latitude-Longitude from each other. Connecting light lines between corresponding native and non-native ranges for each species indicate that legume species have successfully established into novel regions, continents and islands. Note: Lines do not necessarily represent actual dispersal pathways, as some non-native ranges may have been colonized from an intermediate non-native range, rather than directly from the native range.

**Figure 2:**
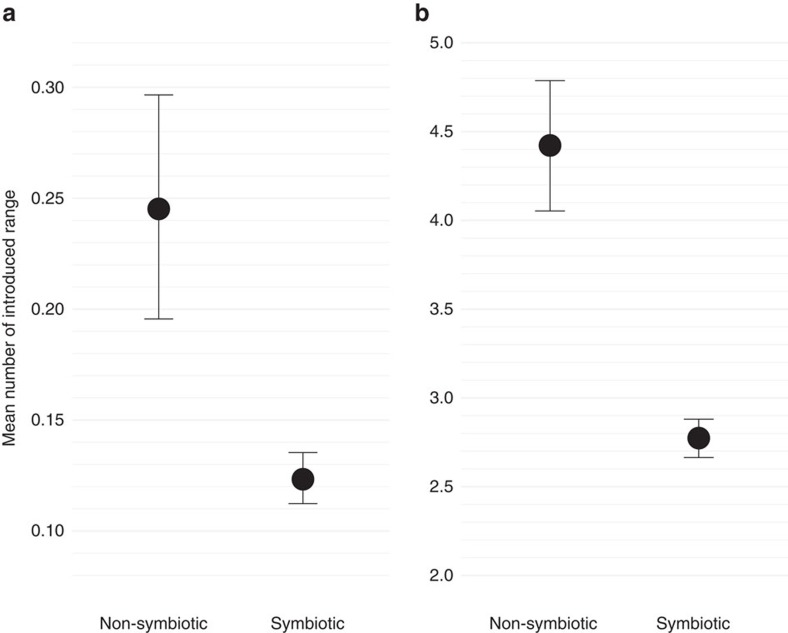
Non-native ranges between symbiotic and non-symbiotic legumes species. (**a**) Mean number of introduced ranges across all legume species studied (including those with no introduced ranges) [(*n*_total_=180,030=(*n*_species_=3,530) × (n_regions_=51)]. (**b**) Mean number of introduced ranges for legume species recorded from at least one non-native region [(*n*_total_=41,412=(*n*_species_=812) × (*n*_regions_=51)]. A species' introduced range is defined by a list of geographic regions of non-native occurrence. Points and error bars represent the mean and 95% confidence intervals from parametric bootstraps, controlling for all covariates (absolute native latitude, total native area, life form, life history, area of non-native region and number of human uses).

Within our dataset, 21.6% of all legume species occur in at least one non-native region (that is, polygon) and 15.8% occur in two or more non-native regions, confirming that many symbiotic and non-symbiotic legume species have successfully invaded or been introduced into new regions, continents and islands (Fig. 1). Our analysis of all species in the dataset at the regional level (see Methods section) show that symbiotic legumes have a significantly lower probability of occurring in non-native regions (Table 1), which translates into 49.7% fewer non-native regions per species (Fig. 2a). Furthermore, for successfully introduced species with at least one non-native range, we found that symbiotic legumes retain a lower probability of occurring in multiple non-native regions (Table 1), translating into 37.1% fewer numbers of non-native regions compared to non-symbiotic legumes (Fig. 2b). We excluded the possibility that non-native symbiotic legume species simply occurred in fewer yet much larger countries (Supplementary Note 1) by confirming that each non-symbiotic species had, on average, a larger total non-native range area (Supplementary Table 1) and no difference in the average size of individual regions that comprise the total introduced range area (Supplementary Table 1).

**Figure 3:**
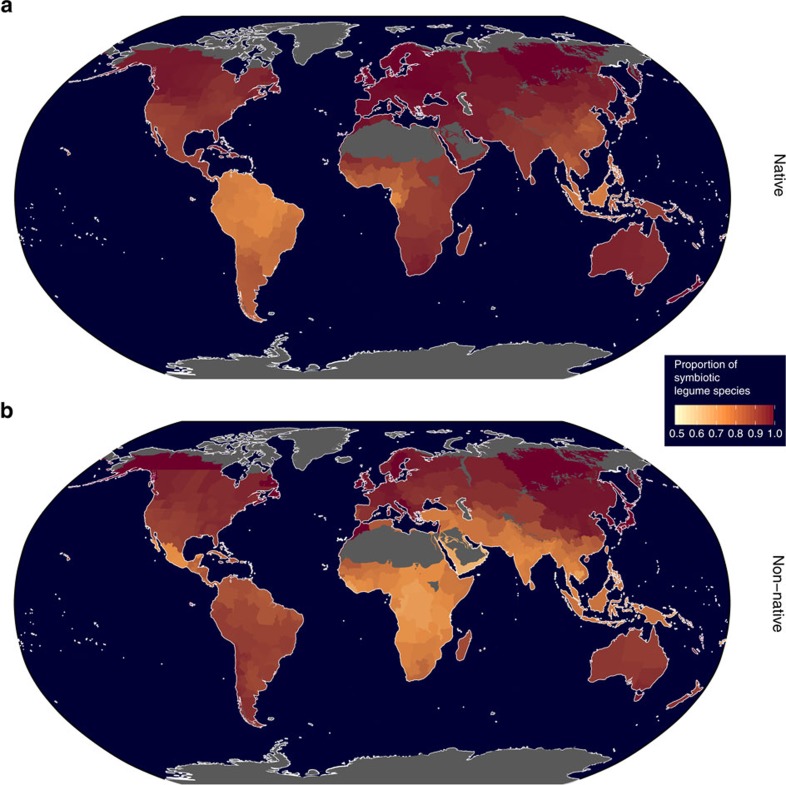
Global proportional distribution of symbiotic legume species. Regions are coloured according to the proportion of symbiotic legume species in (**a**) native and (**b**) non-native ranges. Lighter colours indicate higher proportions of non-symbiotic legume species. Non-symbiotic legume species tend to primarily occur near the equator in their native range. Non-symbiotic legume species currently account for a higher proportion of species within introduced ranges compared to their proportion within native ranges. The figure shows that the increased spread of non-symbiotic legumes spans multiple continents and islands across the world. Grey areas indicate terrestrial ecoregions where legumes are not known to occur^43^. Regions are defined using the Taxonomic Distribution Working Group system^39^.

For species occurring in more than one non-native region we also measured the degree of geographic dispersion between non-native regions and found no difference in the degree of geographic dispersion between either legume group (Supplementary Table 1), showing that introduced symbiotic legumes do not have more or less geographically widespread non-native regions than non-symbiotic legumes. Together, our analyses show that the contrast in introduction success between symbiotic and non-symbiotic legumes was characterised by differences in the number of non-native regions. These results combined support the hypothesis that non-symbiotic legumes have a higher chance of establishing and subsequently spreading to a greater number of geographic areas (Fig. 3).

### Accounting for potentially confounding species traits

We found that latitude of origin, size of a species' native range, plant life form (woody or not woody), life-history (annual or perennial), number of human uses, and the interaction between symbiosis and number of human uses were all significant predictors of introduction success. Non-symbiotic legumes tended to occur more frequently at or near the equator in their native range (Fig. 3; ref. 14). However, our analysis show that latitudinal bias in introduction success favours legume species naturally occurring away from the equator (Table 1), indicating that biased dispersal related to latitudinal effects would favour symbiotic rather than non-symbiotic legumes. Total native area was a significant factor in predicting the prevalence of establishment in non-native regions, (Table 1), but we found no difference in total native range areas between symbiotic and non-symbiotic species (Supplementary Fig. 4).

While woodiness was the dominant life form in non-symbiotic legumes (Supplementary Fig. 4), being woody did not always predict introduction success. Specifically, while woody plants had a higher probability of occurring in non-native regions among all species in the dataset, when the analysis was restricted to introduced species, woody plants had a significantly lower probability of being introduced into multiple non-native regions (Table 1). Furthermore, annual species were more likely to establish in non-native regions (Table 1), but only 0.8% of non-symbiotic species are annual (Supplementary Fig. 4). In total, our analysis show that while total native area, latitude, plant life form and life-history are important (as other studies have also shown^15^), their effects do not eliminate the symbiosis trait as a key determinant of establishment in novel ranges.

The geographic area of a region had no effect on the prevalence of non-native species within it (Table 1). This likely reflects limited variation in area between our regions (Supplementary Fig. 6), combined with other factors being much more important for successful introduction such as the amount of trade a nation receives (this variation due to unknown factors would be reflected in the region-level random effect).

### The role of human use in successful introductions

**Figure 4:**
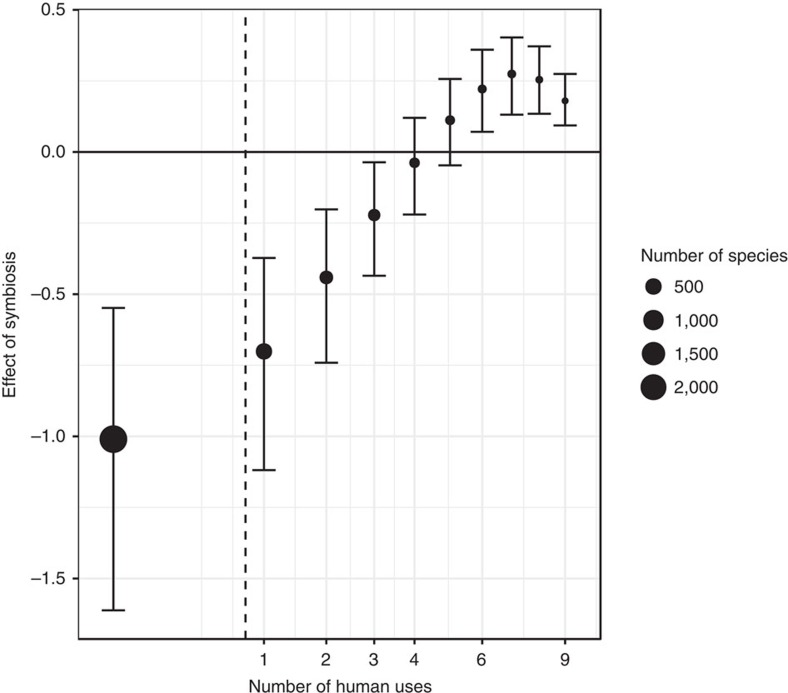
The interaction between symbiosis and human uses. The size of the symbiosis effect on successful introductions at different numbers of human uses, using the full dataset. Dots represent the difference in the predicted number of non-native ranges between non-symbiotic and symbiotic species, standardised by dividing by the predicted number of non-native ranges for non-symbiotic species. The standardisation controls for the differences in the predicted number of non-native ranges between different human use levels (determined by the main effect of number of human uses), and makes the interaction easier to visualize. Error bars represent the 95% confidence interval obtained through parametric bootstrapping. The size of the dots is proportional to the number of species in the dataset with that number of human uses, showing that most species had no uses recorded in the dataset (∼70%). The dotted vertical line represents the mean number of human uses across all species in the dataset (0.77). The x-axis is on a square root scale. Negative values mean the symbiotic species are predicted to have a lower prevalence of non-native ranges relative to non-symbiotic species. Apparent non-linearity is due to logit back-transformation.

We found that ∼30% of species in our dataset had at least one human use, and that legume species with more uses are much more likely to establish in non-native regions (Table 1). Species with human uses may be more likely to establish due to more frequent intentional introduction attempts (i.e., higher human-mediated propagule pressure), which may mask or confound differential establishment patterns driven by the symbiosis trait itself. Our analysis accounts for this potential bias by including it as a covariate (along with its interaction with symbiosis), and then statistically evaluating the main effect of symbiosis at no (that is, zero) human uses (this is important because of the presence of the interaction^16^). The main effect of symbiosis (Table 1) thus evaluates any differences in non-native establishment patterns that are least likely to be impacted by human-mediated propagule pressure. After accounting for the number of human uses in this manner, we found that non-symbiotic legumes are still much more likely to establish in non-native regions (Figs 2 and 4; Table 1).

We also found a significant interaction between symbiosis and the number of human uses, suggesting that human use does have an influence on the successful introduction of symbiotic versus non-symbiotic legumes. We predicted that if human uses were exacerbating the spread of non-symbiotic legumes over symbiotic legumes (above the disparity observed at no human uses) that we should observe a negative interaction in our main model. However, we found a positive interaction, indicating that the disparity between symbiotic and non-symbiotic legumes decrease, rather than increase (Fig. 4). These results combined provide evidence that human-mediated propagule pressure is not generating the pattern that non-symbiotic legumes are more prevalent in non-native regions.

### Influence of phylogenetic history on introduction success

The ability to symbiotically fix nitrogen has been gained and lost multiple times across the legume phylogeny, although the trait is more concentrated in certain clades (Supplementary Fig. 1). If the pattern of successful introductions across species also shows strong phylogenetic structure, it is possible that our results could reflect a lack of independence among the species we used in this study due to their shared evolutionary history with respect to other predictive yet unmeasured traits. However, the probability of having a non-native range has no phylogenetic signal (phylogenetic parameter alpha=31.27; Supplementary Note 2). When we incorporated phylogenetic structure into our analysis, which included all covariates from our main analysis (Supplementary Note 2), non-symbiotic legume species still had a significantly higher probability of establishing in non-native regions (Supplementary Table 2), consistent with results of our main analysis. Overall, these analyses indicate that the increased ability of non-symbiotic legume species to establish in a greater number of non-native ranges was not driven by phylogenetic dependence and makes it unlikely that our results can be explained by another trait that is evolutionarily correlated with symbiosis and also influences introduction success.

## Discussion

In summary, our findings clearly support the argument that nitrogen-fixing legumes are highly dependent on the symbiosis and that this dependency is sufficiently large to generate dispersal or establishment barriers at a global scale across multiple legume species, regions and continents (Fig. 3). Inadequate population density of compatible rhizobia or appropriate environmental conditions for effective nitrogen fixation in introduced ranges are viable explanations for our results. This explanation would suggest that symbiotic legume hosts and their compatible symbionts would frequently need to be introduced simultaneously or that introductions would favour legumes that are able to form associations with a broad diversity of rhizobia^17^,^18^, which is consistent with empirical findings from previous studies examining introduced *Acacia* species^18^,^19^,^20^.

Our results are also consistent with the explanation that an evolutionary investment in the mutualistic interaction with rhizobia has resulted in reduced competitive ability of symbiotic nitrogen-fixing legumes^21^, relative to non-symbiotic legumes. This explanation would require that the fitness cost of harbouring the symbiosis trait is higher in non-native ranges relative to the native range. Changes in soil resource availability have been proposed as a mechanism to alter the cost-benefit ratio of plant nitrogen-fixation^21^ and human activity is often associated with increased nutrient deposition^22^. However, experimental evidence has shown that invasive nitrogen-fixing plants have greater growth in fertile soils compared to invasive co-occurring non-fixing plants^23^, suggesting that symbiotic nitrogen-fixation is a net fitness benefit in non-native ranges, rather than a cost.

Our study also highlights the prominent effects of propagule pressure, mediated by increased intentional introductions associated with human use attributes of species. The number of human uses for a species was a powerful predictor of the prevalence of successful introduced ranges. This is consistent with a number of other studies which found human use to be important in predicting establishment of plant species outside their native range^24^,^25^, including legumes^4^,^26^,^27^. Among highly useful species, we found that the difference in introduction success between symbiotic and non-symbiotic legumes lessens and eventually reverses (Fig. 4). This suggests that if species are highly useful, any natural establishment barriers among symbiotic legumes (that is, lack of mutualist partners) may be overcome through increased human effort to intentionally introduce a species (for example, increased effort to inoculate new sites lacking rhizobia). It is possible that after human intervention has allowed a symbiotic legume species to overcome its establishment barriers, the benefits of nitrogen fixation then allow it to be more successful. However, the majority of species looked at in this study have no recorded human uses, so that the effect prevailing at low human uses is of particular importance for legume species as a whole.

Transplant trials have shown that many legumes rely on soils that are pre-inoculated with compatible microbial symbionts to establish^28^,^29^ and that soils are highly variable in the abundance^30^,^31^,^32^ and inter-continental genetic structure^33^ of compatible rhizobia. However, symbiotic nitrogen-fixation has also been implicated in facilitating the invasion of some of the most widespread and problematic legume species of the world^30^, giving the appearance that compatible rhizobia are cosmopolitan in their distribution. Based on these isolated observations and studies, it has been difficult to establish a general pattern with respect to the role of symbiosis in limiting or facilitating legume establishment^34^. Though the size of our analysis and its global extent is unprecedented, we acknowledge that only a fraction of the estimated ∼19,000 legume species have been characterized for their symbiosis ability (∼20% species and ∼60% of legume genera^35^). Nevertheless, based on an examination of ∼3,500 legume species, our study reveals that symbiotic mutualism traits are important in predicting the introduction success of legumes across multiple continents and islands. Our study further highlights likely ecological costs associated with being a nitrogen-fixing species, and the potential for plant species distributions to be influenced by soil microbial biogeography at a global scale.

## Methods

### Experimental design

The objective of our study was to investigate whether symbiosis with nitrogen-fixing legumes had any predictive power with respect to legume introductions globally. We compiled symbiotic nitrogen-fixation data and geographic distribution data matched to as many legume species as possible (see below). We measured introduction success by counting the number of non-native regions of occurrence for each available species. We then analysed the predictive power of the symbiosis trait on introduction success in the presence of other potentially confounding or correlated covariates that might be important in predicting legume introductions. Once we verified that the significance and direction of response for symbiosis remained after the inclusion of other covariates, we analysed the predictive power of symbiosis, incorporating phylogenetic structure into our analyses.

### Symbiotic nitrogen-fixation data

Nitrogen-fixation status was extracted for all available legume species (members of the family *Fabaceae*) from the publicly available database compiled by Werner *et al*^36^. This database scores each species as either ‘symbiotic' or not, and has been compiled from a number of experimental and observational studies examining the presence or absence of rhizobial infections on 5,427 legume species (see Werner *et al*.^36^ for more details). For the species we used in this study, information on nitrogen-fixation status covers ∼20% of all known legume species (3,530 species out of ∼19,700) and ∼60% of all known legume genera (440 genera out of ∼750). Since we aimed to determine whether symbiosis is a potential barrier or facilitator of initial legume establishment, we evaluated the presence or absence of the ability to symbiotically fix nitrogen as specified in Werner *et al*.^36^; see the original source reference list on figshare^37^ for all references related to every species used in this study.

### Global introduced status data

For each legume species found in the nitrogen fixation database, we searched the International Legume Database and Information Service (ILDIS)^13^ and extracted geographic distribution information using a webscraper written in R^38^. The ILDIS geographic data was compiled by experts who synthesized regional floristic data from the primary literature. Because ILDIS data are a synthesis of recorded locations of living observations and herbarium records by flora experts, they likely encapsulate established species in a given region. In total, 3,973 legume species were found in ILDIS (species not found in ILDIS were discarded from the analyses) that matched the nitrogen-fixation trait database. Species distribution data in ILDIS is coded using the names of hierarchically structured geographic regions (i.e., continent, region, area, and country where available), and indicates whether each region is native, introduced, or unknown, thus capturing dispersal events at both regional and continental scales. We scraped geographic data at all available hierarchies and excluded areas where the introduced status was considered unknown. The geographic names in ILDIS were based on the World Geographical Scheme for Recording Plant Distributions developed by the Taxonomic Database Working Group (TDWG)^39^. We eliminated species records from our dataset just containing ‘unknown' regions, giving a total sample size of 3,530 species. The final dataset used in this study contained 317 non-fixing legumes out of 3,530 (9%), whereas the full Werner *et al*.^36^ database contained 482 non-fixing legumes out of a total 5,427 total legumes (8.9%); therefore, our dataset showed no bias with respect to the proportion of non-fixers relative to all known data on legume nitrogen fixation.

To convert the geographic names in the ILDIS database into usable geographic coordinate data, we downloaded a shapefile containing the standardized TDWG geographic regions as polygons^40^, then matched these polygons to ILDIS geographic names. Where TDWG polygons and ILDIS geographic names could not be matched, we used the next available polygon in the geographic hierarchy (for example, if we could not find a polygon corresponding to an area name, we used the region name instead). Moving up to the next geographic level was only necessary for less than 10% of recorded regions and did not exceed one level. Once all geographic areas were matched to polygons, we only retained those at the lowest available hierarchical scale.

Before analysis we merged any species range polygons that were touching each other and had the same introduction status, to prevent biases in the number of ranges due to finer subdivision within some areas. This way, all final polygons represent non-contiguous ranges.

We checked the accuracy of the polygon occurrences by comparing our polygon data to occurrence records in the Global Biological Information Facility (GBIF)^41^. GBIF gives higher resolution point occurrence records, but for far fewer species (1,830 species) and invasive or introduced status is not recorded for each GBIF record. We found that, on average, ILDIS polygons for each species captured 93% of GBIF points (see Supplementary Fig. 2 for some example species maps). For points that fell outside ILDIS polygons, we calculated the geographic distance to the nearest ILDIS polygon and found that most points were geographically very close to an ILDIS polygon, with no obvious bias in the distribution of geographic distances between symbiotic and non-symbiotic legumes (Supplementary Fig. 3). There was no obvious differences in the presence or number of ‘unknown' introduction status polygons between symbiotic and non-symbiotic species (Supplementary Fig. 4), indicating that any ambiguities in polygon status was not different between our legume comparison groups. Together, these indicate that ILDIS geographic range data was reliable.

### Other species trait data

We also scraped plant life-form (woody or not woody), life-history (annual or perennial), and information on human uses from ILDIS, as previous invasion studies have found these to be important factors in predicting legume invasion success^15^. We were able to obtain plant life form for 3,500 legume species and life-history for 3,462 legume species. We converted life form and history data into two binary traits by coding life form as a 1 for species that were woody (trees or shrubs), and as 0 for non-woody (herbs). Likewise, for life history, species that had an annual life history were assigned a value of 1, and perennial species assigned a 0.

Life history and life form data was unavailable for ∼500 species, which would lead to a fairly large reduction in our sample size when including this data as covariates. To maintain full power in our models (see next section for model details), while still accounting for the potentially confounding effects of life form and life history, we imputed the missing values in these traits. We did this using a simple taxonomic imputation. If there were other species in the same genus as a missing species, its value was assigned to be the mean of the life form or life history values for that genus (for example, a value of 0.9 for life form would be assigned to a species in a genus with 90% woody species). If the data were missing for an entire genus, we used the mean of all species found in the same tribe in the same manner. Therefore, the life form and life history variables can be interpreted as the probability that a species is woody or annual respectively, based on their taxonomic group. 83 and 84.6% of missing species were filled at the genus level, for life form and life history respectively, and the remaining missing species at the tribe level. Some genera contained a mixture of either trait value, but 87.5 and 91.2% of genera could be coded greater than 0.75 or less than 0.25 (for example, more than 75% of the genus was one trait or the other) for life-form and life-history respectively. Most genera that contained species with missing data were entirely of one life form (73%) or life history (77%) and could be coded unambiguously as 0 or 1 based on their genus grouping. We repeated our main analysis with two other trait imputation methods (see Supplementary Note 3) and our results did not change qualitatively, with all model coefficients changing only superficially, and no change in levels of significance for any factor, indicating that our results are robust to several methods of imputation. Therefore, only the results using the first method described above (that is, taxonomic mean imputation) are reported.

Human use data was recorded in ILDIS by specifying whether a species was known to have a use in any of 11 different use categories (Chemical products, Domestic, Environmental, Fibre, Food and Drink, Forage, Medicine, Miscellaneous, Toxins, Weed or Wood). If none of these categories was specified in ILDIS, we assumed the species had no known uses. We calculated a human use covariate by counting the number of known human use categories for each species to create a value which could range from 0 to 9 (no species in our study had all 11 use categories), which we refer to here as ‘number of human uses'.

### Statistical analysis

We modelled the prevalence of successfully introduced species in regions across the world using a generalised linear mixed model (GLMM).

Under the TDWG scheme, there are four nested levels of geographic range specification, from smallest to largest: the country, area, region, and continent level (though the country level does not necessarily always correspond to political countries). In our data, after translating from ILDIS to TDWG, geographic range was determined to different degrees of resolution, depending on the species. Some but not all species were specified at the TDWG area level, for example. To make all species comparable, we analysed introduced ranges on a single level. All non-native ranges were specified to at least the TDWG region level or below, so we used this as our focal geographic unit for the range during our analysis. Henceforth, we will refer to what TDWG calls region level simply as ‘regions'. There were 51 regions in total (see Supplementary Fig. 6 for a map of the regions used in this part of the analysis).

Our response data therefore is made up of a vector of zeroes and ones *y*_*ij*_, which could be arranged into a matrix of *n*_species_ rows, and *n*_region_ columns *Y*_*ij*_ containing a one if species *i* is found non-natively in region *j* and a zero if it is not (e.g., the species is coded as one if at least one of its non-native polygons fell in the region). We were interested in testing whether symbiosis affects non-native prevalence across the globe, while controlling for several potentially biasing factors. Our model is the following:

The non-native presence of a species *i* in region *j* is modelled as a realization of a binomial process on the probability of species *i* in region *j*, *P*_*ij*_:


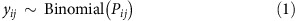


The probability of species *i* in region *j* is a function of the symbiosis status of species *i* (*SS*_*i*_; equals 1 if symbiotic, 0 if non-symbiotic), a number of potential covariates (*x*_*ik*_ and *x*_*jk*_), and random effects for species (SP[*i*]) and region (RE[*j*]):


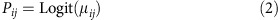



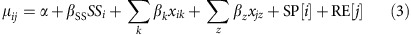



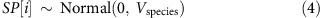



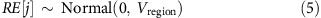


Where *α* is an intercept term, *β*_SS_ is the fixed effect coefficient determining the effect of symbiosis, *β*_*k*_ is the fixed effect coefficient determining the effect of species-level covariate *k*, *β*_*z*_ is the fixed effect coefficient determining the effect of region-level covariate *z*, and *V*_species_ and *V*_region_ are the variance parameters for the species and region random effects, respectively.

We also initially included the TDWG continent level region as a higher level random effect (within which region was nested), however, we removed it in our final analysis as it explained almost no variation in the model (that is, variance parameter was very close to zero).

This mixed effects model with crossed random effects has a number of advantages over a simpler species-level analysis. First, it allows the inclusion of both region-level as well as species-level covariates. Second, the random effect for region accounts for spatial non-independence within regions of the world. In a species-level only analysis, we would not be able to say if our results were driven just by one or a few regions across the globe, whereas with the full mixed model, our inferences are applicable globally.

Species-level covariates (*x*_*k*_) included in the model were: the absolute latitude of the centroid of a species' native range polygons, the total area of the species' native range polygons, the species' two binary life history traits (woody or not woody, and annual or perennial), and the number of human uses of the species according to ILDIS. We also included a symbiosis by number of human uses interaction, because we hypothesized that the number of human uses would reflect the probability of a species being deliberately introduced (as opposed to unintentionally), and this may affect the strength of any biological factors on introduction probability.

We included one region level covariate (*x*_*z*_): the area of the introduced regions. This was to control for the possibility that larger areas may be more likely to have more introduced species simply due to a sampling effect.

To fit the model, we used the lme4 package in R^42^. When fitting the model, all continuous covariates were mean centred (subtracted the mean such that zero corresponds to the mean) and scaled by the standard deviation, except for the number of human uses, for which zero is a biologically meaningful value, corresponding to the state at which deliberate introduction attempts should be lowest, and thus acting as the best reference point at which to evaluate other effects (see Frasier^16^ for useful discussion).

We ran two versions of the above model. The first model included all data on all species. Given the excess number of zeroes present in our analysis (that is, ∼78% species only occur natively), we ran a second model only on the species occurring in at least one non-native region to confirm similar results when zeroes were removed from the dataset.

All response variables were analysed in R^38^ and included the symbiosis trait, latitude of origin, total native area, plant life-form (woody or not woody), plant life-history (annual or perennial), number of human uses, the interaction between symbiosis and number of human uses, and the area of the introduced region as predictors. We calculated the correlation between all of our species-level model predictors (Supplementary Fig. 5) and the highest correlation occurred between being woody and annual in our first (r=−0.49) and second (*r*=−0.59) model (Supplementary Fig. 5).

### Testing the model effects

We tested whether symbiosis and covariates were significant predictors of the prevalence of successful species introductions using parametric bootstrapping. We simulated 1,000 new response vectors (*y*_*ij*_) from the fitted model, using observed values of the fixed effect variables, and fixing species and region random effects at their estimated values. For each bootstrapped response vector we refit the same model to it and collected the fixed effect coefficients. We then calculated the 95% confidence intervals by determining the 0.025 and 0.975 quantiles of each coefficient's bootstrapped sample (Table 1). A fixed effect was classified as significant if its 95% confidence interval did not overlap zero. We also calculated 99 and 99.9% confidence intervals (Table 1).

### Visualising the results

To translate the model results into a set of more intuitive measures for display, we again used parametric bootstrapping. To show the effect of symbiosis on the prevalence of successful introduced ranges, after controlling for all covariates, we simulated 1,000 response vectors from the fitted model, but this time setting all covariates to a value of zero (symbiosis remained at its observed values). This procedure removes the variation explained by the covariates in a way analogous to least square means in a standard statistical analysis.

We took these 1,000 sampled response vectors and calculated several summary statistics for plotting. For each bootstrap sample, we calculated the mean number of introduced ranges for symbiotic and non-symbiotic species. We then plotted the mean and 95% confidence intervals of these values based on the bootstrap samples (Fig. 2).

### Data availability

The full data set, including cleaned up ILDIS data, nitrogen-fixation data, and all covariates, is available from the authors on request. All data in their original form are available from public repositories (see Methods).

## Additional information

**How to cite this article:** Simonsen, A. K. *et al*. Symbiosis limits establishment of legumes outside their native range at a global scale. *Nat. Commun.* **8,** 14790 doi: 10.1038/ncomms14790 (2017).

**Publisher's note:** Springer Nature remains neutral with regard to jurisdictional claims in published maps and institutional affiliations.

## Supplementary Material

Supplementary Information Supplementary Figures, Supplementary Tables, Supplementary Notes and Supplementary References

## Figures and Tables

**Table 1:** Introduction success as predicted by the symbiosis trait.

**Factor**	**All species**			**Non-native species Only**		
	**Coefficient**	**Lower CI**	**Upper CI**	**Coefficient**	**Lower CI**	**Upper CI**
Intercept	−8.055	—	—	−3.125	—	—
Symbiosis?	−0.523†	−0.960	−0.437	−0.587†	−0.832	−0.416
Latitude	0.142†	0.094	0.210	0.026	−0.013	0.063
Total native area	0.194†	0.119	0.211	−0.058†	−0.092	−0.038
Annual?	1.092†	0.915	1.267	0.198†	0.065	0.329
Woody?	0.353‡	0.084	0.404	−0.476†	−0.634	−0.408
Number of human uses	0.964†	0.865	0.981	0.323†	0.282	0.370
Area of introduced region	0.010	−0.049	0.046	0.019	−0.046	0.051
Symbiosis by human uses interaction	0.152†	0.104	0.223	0.080†	0.042	0.129

CI, 95% confidence interval.

Symbiosis is incorporated into the model as the presence or absence of the trait, with the inclusion of other factors found to predict introductions in our legume dataset. A negative coefficient indicates lower introduction success for symbiotic legumes compared with non-symbiotic legumes. We excluded non-significant interaction terms. 95% confidence intervals were obtained from parametric bootstrapping. Each response variable is modelled at the species by region level, and the model included a species and a region random effect to account for non-independence of observations within species or regions. The estimated variance for the species and region random effects respectively were 4.83 and 0.85 for the all species model, and 0.9 and 1.43 for the non-native species only model.

^†^99.9% CI does not overlap zero.

^‡^99% CI does not overlap zero.
