# Supplementary material for: Symbiosis limits establishment of legumes outside their native range at a global scale
Source: Nat Commun. 2017 Apr 7;8:14790. doi: 10.1038/ncomms14790 (PMC5385628; doi:10.1038/ncomms14790)
Supplement: Supplementary Information — Supplementary Figures, Supplementary Tables, Supplementary Notes and Supplementary References [file ncomms14790-s1.pdf]

Supplementary Note 1: Analysis of geographic properties of non-native ranges

Our main analysis establishes that non-symbiotic legume species are more likely to become successfully established in non-native ranges. However, it is possible that symbiotic and non-symbiotic legumes could differ in the geographic attributes of those non-native ranges due to the inherent limitations of TDWG politically based range areas. In this supplement, we include analyses of several measures of geographic properties of legume species' non-native ranges, to test the possibility that any introduced patterns are dominated by 'political' signal (i.e. size of a country) rather than biological signal. For example, if symbiotic legumes tended to occur in larger individual non-native countries (though fewer).

Here we analyse three geographic variables at the species level: geographic dispersion, total range area, and mean range area of non-native ranges. To account for any potential bias in spatial polygon (i.e. range) clustering between symbiotic and non-symbiotic legumes with two or more non-native polygons, we calculated a variable measuring the degree of geographic dispersion of a species' non-native range by calculating the mean distance between the centroid points of each non-native polygon to the centroid of all of the non-native polygon's centroid points. This value increases as the average distance between ranges increases. Distance to centroid values were calculated using great circle distances approximated by the geosphere package in R[1]. This measure was square root transformed prior to analysis.

We also calculated the log-transformed total area of all non-native ranges for each species, as well as the log-transformed mean area per individual non-native range polygon. All the following analyses were performed only on the subset of species for which there was at least one non-native range polygon (zeroes were not included), or at least two non-native ranges in the case of geographic dispersion, and analysed using standard Gaussian linear models (after appropriate transformations of the response variables).

The mean area of non-native polygons and their geographic dispersion was not significantly different between symbiotic and non-symbiotic species (Supplementary Table 1). Total area of non-native polygons, however, was significantly smaller for symbiotic species as compared with non-symbiotic species. This is consistent with our main result, in that it suggests that not only do symbiotic species have a lower prevalence of non-native ranges (Supplementary Table 1), but they also have a smaller total area of introduction once they have been introduced. We recognize the caveat of interpreting area measures from TDWG political regions. But our analyses of geographic properties indicate that any variation introduced by using TDWG definitions do not conflict with biological inferences obtained from our main results.

Supplementary Table 1. Regression of various geographic measures of successful non-native ranges (with denoted response variable transformations) against the presence or absence of the symbiosis trait, with the inclusion of other factors found to predict successful introduction in our legume dataset. A negative coefficient indicates a lower response value for symbiotic legumes compared with non-symbiotic legumes. Each cell contains the estimate with 95% confidence interval following in brackets.

	Dependent variable:		
	**Total Area of Non-native Ranges (log)**	**Mean Area of Non-native Ranges (log)**	**Dispersion of Non-native Areas (sqrt)**
**Intercept**	**28.079**	**26.975**	**2,153.489**
**Symbiosis?**	**-0.942* (-1.770, -0.113)**	**-0.566 (-1.221, 0.089)**	**-265.839 (-567.497, 35.820)**
**Native Latitude**	**0.346*** (0.185, 0.507)**	**0.392*** (0.264, 0.519)**	**41.058 (-13.298, 95.415)**
**Total Native Area**	**-0.255*** (-0.379, -0.131)**	**-0.221*** (-0.319, -0.123)**	**65.836** (24.733, 106.939)**
**Woody?**	**-0.723** (-1.186, -0.260)**	**-0.478* (-0.844, -0.113)**	**-111.946 (-273.083, 49.192)**
**Annual?**	**0.685* (0.109, 1.260)**	**0.621** (0.166, 1.076)**	**312.930** (111.044, 514.817)**
**Number of Human Uses**	**0.437*** (0.231, 0.642)**	**0.142 (-0.020, 0.304)**	**50.123 (-19.663, 119.908)**
**Symbiosis X # of Human Uses Interaction**	**0.114 (-0.107, 0.334)**	**0.080 (-0.094, 0.254)**	**55.763 (-18.989, 130.516)**
Note:			***<0.05,**p<0.01,***p<0.001**

Supplementary Note 2: Accounting for phylogenetic non-independence

There is a possibility that phylogenetic non-independence could influence patterns of non-native establishment of symbiotic nitrogen-fixation in our analyses, so we performed several tests to try and rule this possibility out. To do so, we used the most complete angiosperm phylogeny available from Zanne *et al*.[2,3]. We dropped all the tips of the publicly available Zanne *et al*. phylogeny[2,3] that were not found in our legume database, giving a total of 1301 legume species available (1140 symbiotic species, 161 non-symbiotic species) for phylogenetic analyses. This is a significant reduction in our sample size (nearly ⅓), but assuming a relatively random sample, we should be able to generalize our results here to the full dataset.

We were unable to create an exactly analogous model to our main result (i.e. hierarchical mixed model), because there is no available software that can feasibly analyse a dataset of this size while including both phylogenetic structure and crossed random effects to account for regional non-independence. Therefore, we used a species-level analysis comparing species that had at least one non-native range to species with no non-native ranges. We modelled the probability that a species had at least one non-native range using phylogenetic logistic regression implemented in the R package phylolm[4]. This method uses maximum penalized likelihood, which maximizes the penalized likelihood of the logistic regression using Firth's correction, which is implemented in the phyloglm function (model parameter = "logistic_MPLE")[4]. We used the 95% confidence interval generated by a bootstrap procedure with 1000 replicates (boot = 1000 in phyloglm) to determine if a coefficient was different from zero.

We ran two models. The first used no independent variables and was designed to test if there was any significant phylogenetic signal in the probability of a species having a non-native range. If there is no phylogenetic signal in the response it is unlikely that phylogenetic non-independence could affect our main result. However, it is still possible that phylogenetic signal could be obscured by the effects of covariates. So additionally we ran a model including the same set of independent variables used in our

main model (symbiosis, native range area, native latitude, life history, life form, number of human uses, and symbiosis by number of human uses interaction). If we see similar results of the symbiosis effect in this model (which explicitly accounts for phylogenetic signal) to our main results, this would be additional confirmation that phylogenetic signal is unlikely to affect our main results using the full dataset.

The first phylogenetic model with no independent variable estimated a phylogenetic parameter alpha of 31.27. The larger the phylogenetic parameter the faster phylogenetic correlations decay and the less phylogenetic signal there is. Ives and Garland[5] showed that an alpha parameter of 1 corresponds approximately to a level of phylogenetic correlation equivalent to what we might expect in a pure Brownian motion model. Therefore, the phylogenetic signal in the probability of having a non-native range decays at roughly 31 times what we expect under Brownian motion. In other words, there is very little phylogenetic signal in this trait, suggesting we likely do not have to worry about the influence of phylogeny on our main result.

The second phylogenetic model gave results very similar to our main results. Most importantly the symbiosis effect was negative and its confidence interval did not overlap zero (Supplementary Table 2). The only difference we saw in this model from our main results was that the symbiosis by number of human uses interaction was not significant, whereas in our main result we found a significant positive interaction. This could be due to the large decrease in sample size in this model or the slightly different formulation of the model (e.g. lack of a geographic area parameter or using probability of at least one non-native range rather than the prevalence of non-native ranges). The low estimated alpha in this model compared with the first model implies that the covariates included in the model have induced phylogenetic signal in the residuals. Nevertheless, the factors of interest are still in the direction expected based on our main results. This means the phylogenetic signal is either not strong enough to obscure the symbiosis pattern, or it actually acts in a direction that strengthens the result we found. Overall these results suggest that phylogenetic independence does not eliminate the results of our main analysis, where we were unable to include phylogeny.

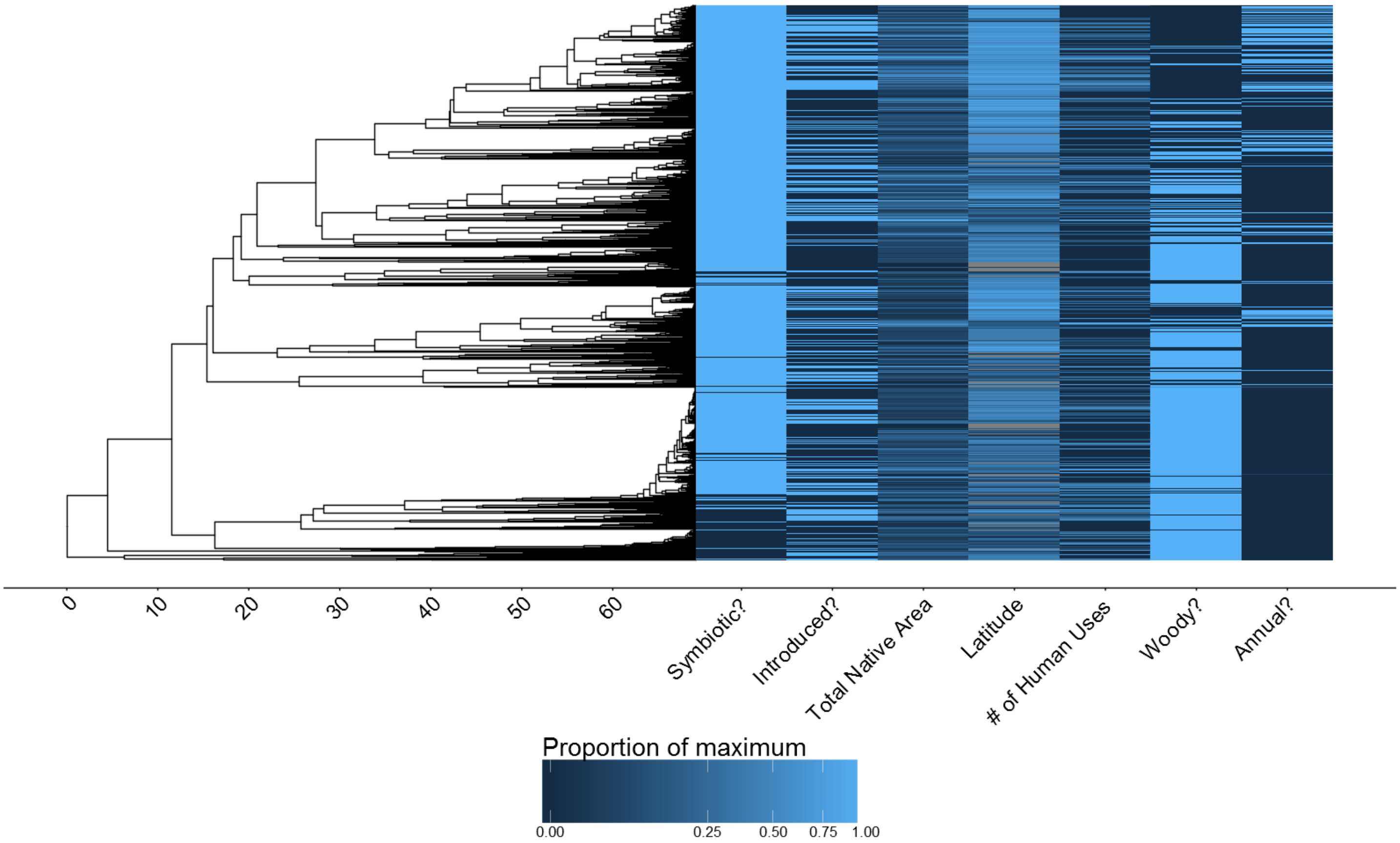

Supplementary Figure 1. Phylogeny of legumes with introduction status, symbiosis traits, and covariates mapped on. Raw trait values are presented as standardized values between 0 and 1, calculated by dividing by the maximum trait value. “Symbiotic?” refers to whether the species is a nitrogen fixer or not, with a value of 1 (blue) representing yes, and 0 (black) representing no. “Introduced?”, “Woody?”, and “Annual” are similarly binary. Grey indicates missing data. Numbers on the x axis represent branch-lengths in units of millions of years since the root of the legume phylogeny.

Supplementary Table 2. Phylogenetic logistic model showing effect of symbiosis and covariates on the probability of being having a non-native range. The phylogenetic (alpha) parameter measure the strength of phylogenetic correlation, with lower values corresponding to stronger phylogenetic signal[5]. CI for phylogenetic parameter not shown because it is restricted to be greater than zero.

Factor	Coefficient	Lower CI	Upper CI
Phylogenetic Parameter (alpha)	0.057	-	-
Intercept	-2.638	-	-
Symbiosis?	-0.672**	-1.220	-0.157
Latitude	0.217*	0.043	0.370
Total Native Area	0.206***	0.086	0.333
Annual?	0.519**	0.098	0.874
Woody?	0.734**	0.256	1.177
Number of Human Uses	0.956***	0.726	1.238
Symbiosis by Human Uses Interaction	-0.043	-0.352	0.224

* 95% CI does not overlap zero

** 99% CI does not overlap zero

*** 99.9% CI does not overlap zero

Supplementary Note 3: Imputation of missing trait values

To determine whether our results were sensitive to the method of imputing missing life history and lifeform traits, we also tried two other imputation methods. The two other methods were a 'majority rules' taxonomic imputation, and an imputation based on other species traits using Random Forest.

For the second method, or majority rules taxonomic imputation, we assigned a state to each trait in the same manner as for the taxonomic mean imputation used in the main text, except instead of using the mean for a taxonomic group, we used the mode, or the most common value. This way, each trait could only take on values available in the original dataset (a 0 or a 1).

For the third method, we used Random Forest to build a predictive model of each trait based on the observed values of other traits, using those observations that that had no missing data (e.g. a random forest classification model predicting life form from latitude, native range area, life history or whatever was available.) This model was then used to predict the values where observations were missing (again, only 0 or 1 values could be produced using this method). This was carried out by the 'missForest` package in R[6]. Briefly, the 'missForest' package uses an iterative algorithm to predict values of a missing variables from non-missing values in other variables. It starts with the variable with the fewest missing values, then fills the values in using randomForest predictions using all other variables as predictors. This imputation process repeats for the variable with the next fewest missing values, and so on until all variables are imputed. It then repeats the entire procedure (because missing value imputation in later variables could change the predictions for earlier variables) until a stopping criterion is met. This stopping criterion is based on convergence of the final imputed dataset, that is, the algorithm stops when the difference between the newest version of the dataset and the previous version is no longer decreasing (see Stekhoven and Bühlmann[6] for more details of the procedure).

Our results from the main model analyses did not change qualitatively regardless of which imputation method we used, with all model coefficients changing only superficially, and no change in levels of significance for any factor, indicating that our results are robust to the method of imputation chosen.

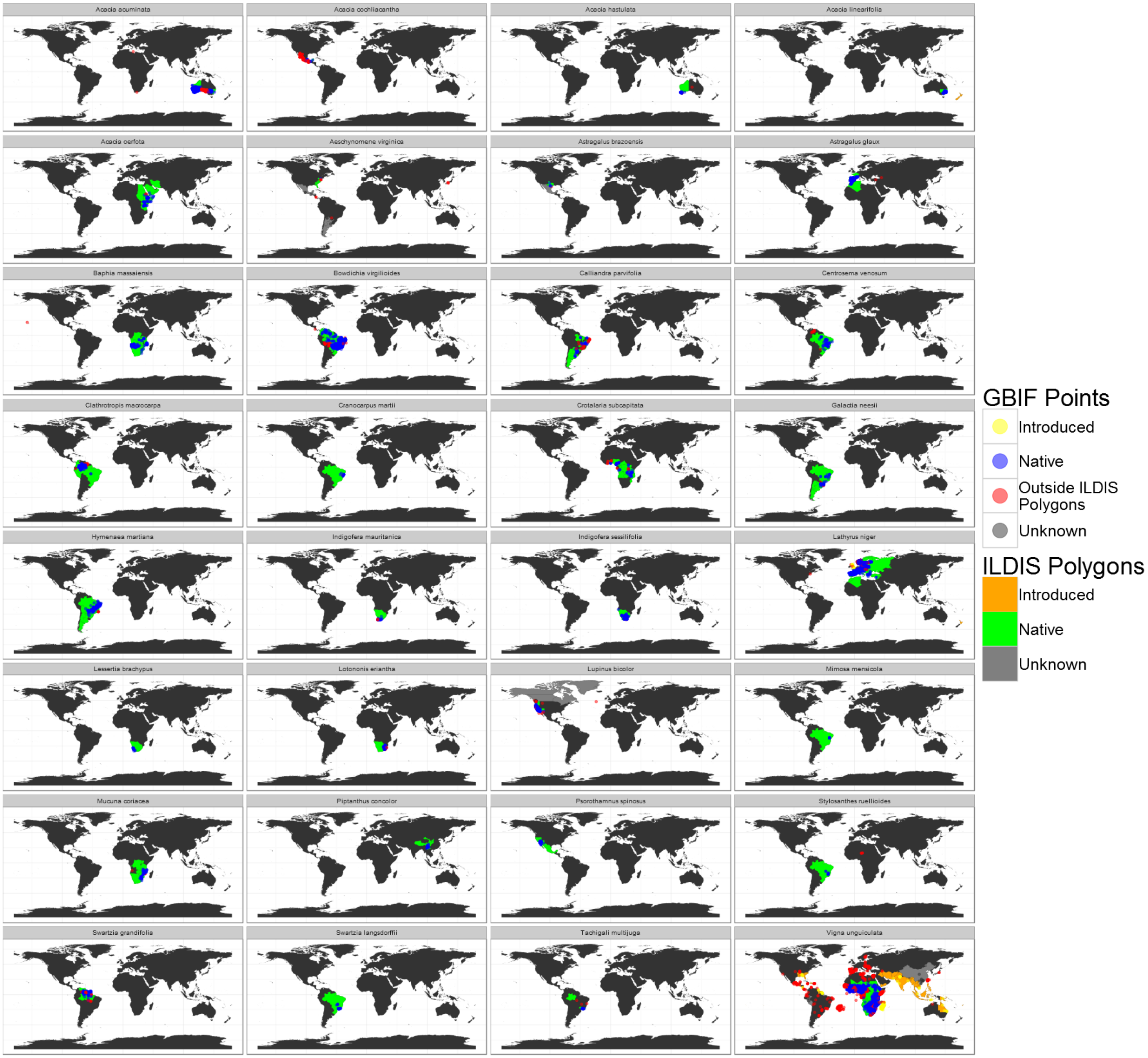

Supplementary Figure 2. Example range species distribution maps for 32 randomly chosen species with both ILDIS and GBIF data. Native (green), introduced (orange) and unknown status (grey) ranges are defined as polygons according to TDWG World geographic scheme of plant distributions. Overlaid occurrence records from GBIF generally show the degree of consistency between ILDIS and GBIF. GBIF points that fall inside ILDIS polygons are coloured according to the status of the polygon they fall in (blue = native, yellow = introduced, grey = unknown); those that fall outside ILDIS polygons are coloured red. Most GBIF points that fall outside ILDIS polygons are not far from ILDIS polygons (see also Supplementary Fig. 3).

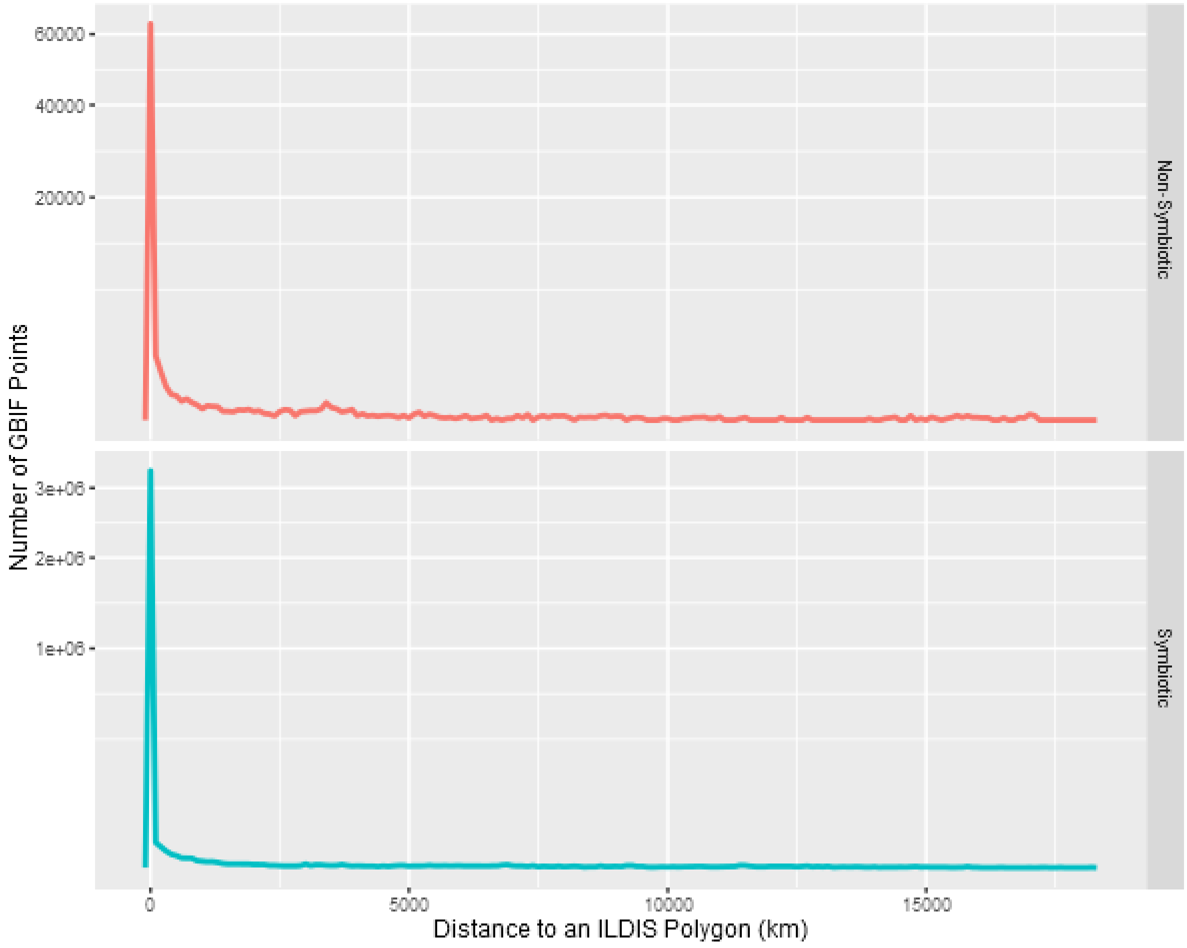

Supplementary Figure 3. Distribution of geographic distance to the closest ILDIS polygon for GBIF records (including zero distance when the GBIF record was inside an ILDIS polygon).

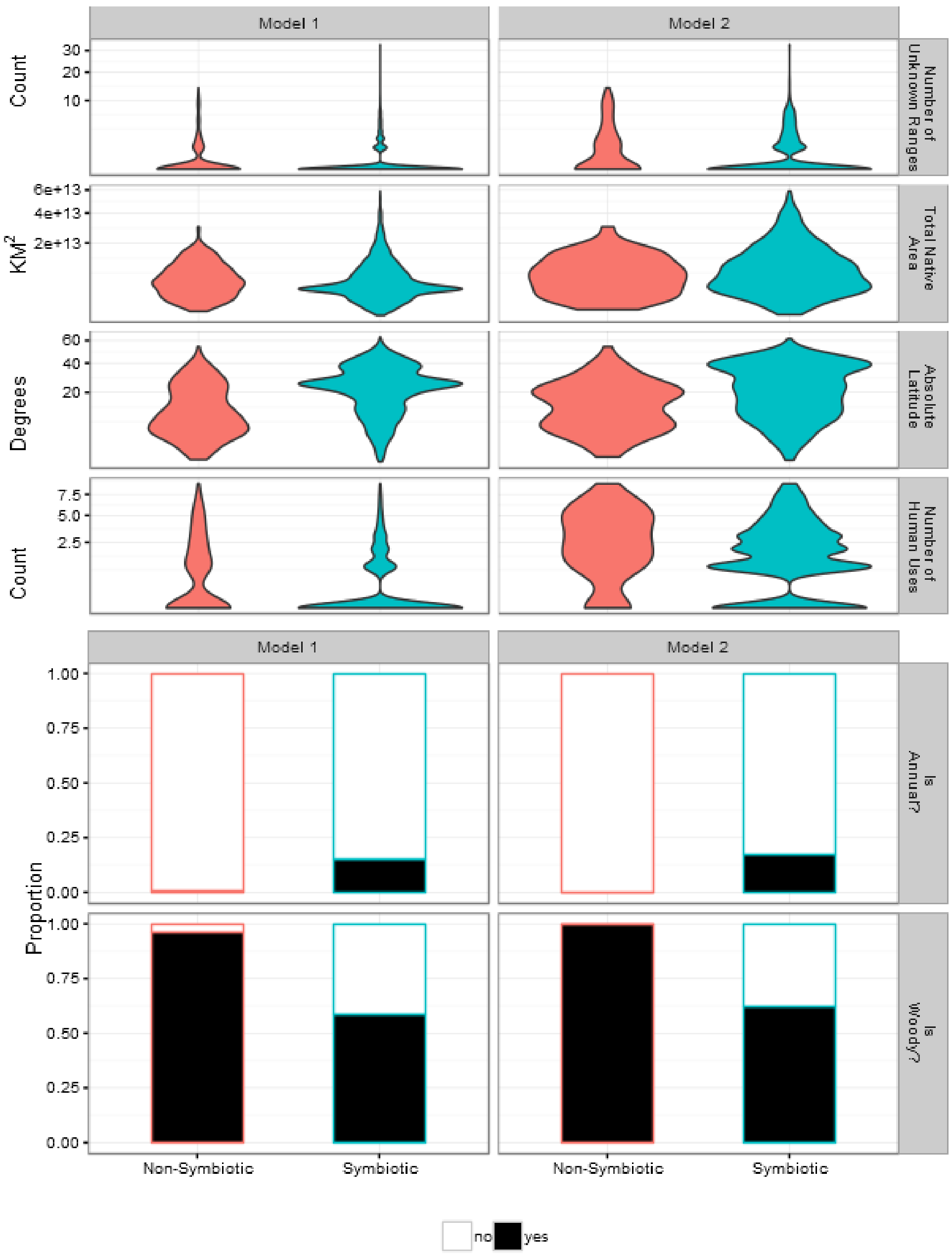

Supplementary Figure 4: Differences in trait or factor values between symbiotic and non-symbiotic legumes. The top panel shows the distribution of the number of unknown ranges for symbiotic and non-symbiotic legumes, showing there is no bias in the reliability of the ILDIS database with respect to the symbiosis. The remaining five panels show the distribution of the trait covariates we used in the statistical models. The top three panels are violin plots: the width of the shape at a given y value is proportional to the density of points there. The bottom three panels plot trait data as stacked bar charts, with black corresponding to the proportion of "yes" state, and white to the proportion of the "no" state. The left and right columns show the data used in our first and second models respectively, Model 1 being the full dataset, and Model 2 being the reduced dataset including only species with at least one introduced range.

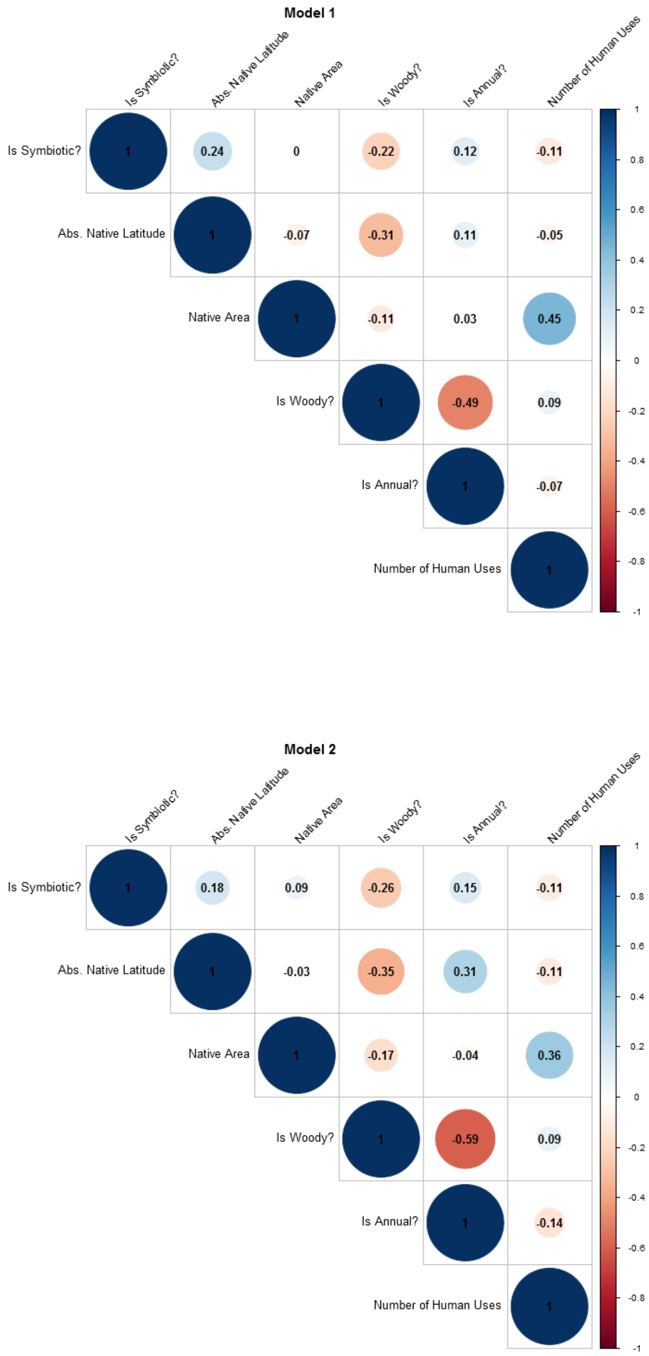

Supplementary Figure 5: Correlation Matrix for all model predictors. Pearson correlation coefficients are shown. For plant life history, annual was coded as 1 and perennial as 0. For plant life form, wood was coded as 1 and non-woody as 0. Human use is indicated by the number of uses for each species. For symbiosis, symbiotic legumes were coded as 1 and non-symbiotic as 0. Abs native latitude stands for absolute native latitude and native area is the summed area of all native countries, regions or states for each species. The top and bottom panels show the data used in our first and second models respectively, Model 1 being the full dataset, and Model 2 being the reduced dataset including only species with at least one introduced range.

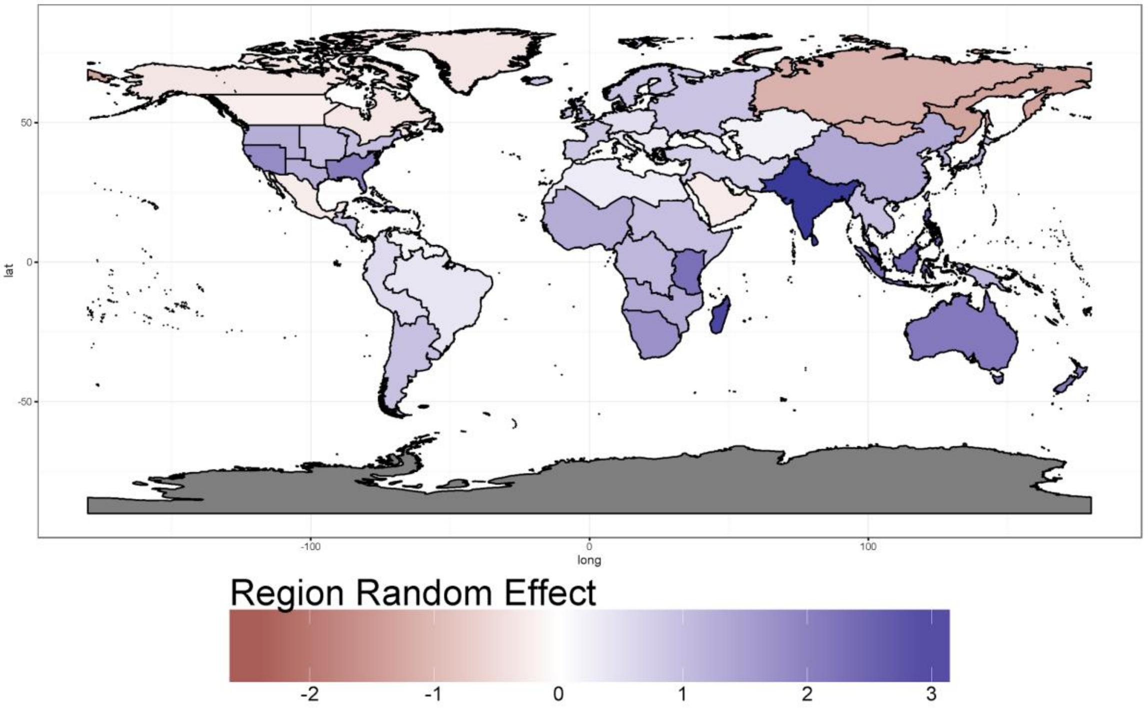

Supplementary Figure 6. Map showing regions used in main analysis. If a species had at least one non-native polygon in a region it was considered present, and absent otherwise. Shading indicates the random effect of region in main model 1 for the number of non-native species in the region. This reflects regional variation in the number of non-native species remaining after accounting for other factors in the model. Blue colours represent regions where there were more non-native species than predicted, and red colours where there were fewer. As expected, this mirrors what is apparent in Figure 1 from the main text, as far as where non-native species are most and least prevalent.

Supplementary References

1. Hijmans, R. J. *et al.* geosphere: Spherical Trigonometry (2015).
2. Zanne, A. E. *et al.* Three keys to the radiation of angiosperms into freezing environments. *Nature* **506,** 89–92 (2014).
3. Zanne, A. E. *et al.* Data from: Three keys to the radiation of angiosperms into freezing environments. (2013). doi:10.5061/dryad.63q27.2
4. Ho, L. S. T. & Ane, C. A linear-time algorithm for Gaussian and non-Gaussian trait evolution models. *Systematic Biology* **63,** 397–408 (2014).
5. Ives, A. R. & Garland, T., Jr. Phylogenetic logistic regression for binary dependent variables. *Syst. Biol.* **59,** 9–26 (2010).
6. Stekhoven, D. J. & Bühlmann, P. MissForest--non-parametric missing value imputation for mixed-type data. *Bioinformatics* **28,** 112–118 (2012).
